# Pattern Matters in the Aposematic Colouration of *Papilio polytes* Butterflies

**DOI:** 10.3390/insects15070465

**Published:** 2024-06-22

**Authors:** Huile Lim, Ian Z. W. Chan, Antónia Monteiro

**Affiliations:** Department of Biological Sciences, National University of Singapore, 16 Science Drive 4, Singapore 117558, Singapore

**Keywords:** aposematism, *Papilio polytes*, imperfect mimicry, pattern, colour

## Abstract

**Simple Summary:**

Many toxic animals display bright colour patterns to warn predators about their toxicity. Their colour patterns are sometimes mimicked by other non-toxic organisms to evade predation. These mimics, however, may not match their model organisms perfectly. It is unclear how much their colour patterns can vary away from the model before they become ineffective. In this study, we investigated how predation risk of the non-toxic Common Mormon butterfly (*Papilio polytes*) is affected by two progressive modifications of its wing pattern that make it more distinct from its toxic model, the Common Rose (*Pachliopta aristolochiae*). In the first modification, we exchanged the position of the red and white colour patches but kept the overall pattern constant. In the second modification, we created an eyespot-like shape from the pre-existing pattern elements by moving their positions in the wing, altering the overall wing pattern. We deployed butterfly paper models in the field, where all models displayed the same colours but had different patterns. Both modifications increased attack risk by predators relative to wildtype patterns, with the eyespot-like modification having the highest predation risk. Our results show that avian predators can distinguish between all three patterns tested, and that predators learn to avoid colours, not in isolation, but as part of specific patterns.

**Abstract:**

Many toxic animals display bright colour patterns to warn predators about their toxicity. This sometimes leads other sympatric palatable organisms to evolve mimetic colour patterns to also evade predation. These mimics, however, are often imperfect, and it is unclear how much their colour patterns can vary away from the model before they become ineffective. In this study, we investigated how predation risk of the palatable Common Mormon butterfly (*Papilio polytes*) is affected by two alterations of its wing pattern that make it progressively more distinct from its model, the Common Rose (*Pachliopta aristolochiae*). We deployed butterfly paper models in the field, where all models displayed the same colours but had different patterns. In the first modification from the Wildtype pattern, we exchanged the position of the red and white colour patches but kept the overall pattern constant. In the second modification, we created an eyespot-like shape from the pre-existing pattern elements by moving their positions in the wing, altering the overall wing pattern. Both modifications increased attack risk from predators relative to Wildtype patterns, with the eyespot-like modification having the highest predation risk. Our results show that avian predators can distinguish between all three patterns tested, and that pattern is important in aposematic signals. Predators learn to avoid aposematic colours, not in isolation, but as part of specific patterns.

## 1. Introduction

Aposematism is a two-pronged strategy which combines a noticeable feature that aids in predator learning with some form of unprofitability, such as a chemical defence, that deters the same predator from attacking an organism with identical or similar features in the future [1]. Taking advantage of these aposematic signals, Batesian mimics [2,3] are palatable species that mimic the appearance of unpalatable model species to deter predation attempts on themselves. The presence of similar noticeable bright colours between models and mimics is widespread [4,5,6,7,8] and found in a wide range of organisms, including, but not limited to, fish, poison frogs, and snakes [9,10,11,12,13,14]. Stevens and Ruxton [15], however, highlight a gap in the understanding of how pattern per se, or the spatial positioning of colour patches across a colour pattern, affects mimicry and why mimics do not always perfectly resemble their models [13,16,17,18,19,20]. 

The benefit of accurate mimicry is thought to be more effective protection against potential predators [21]. But imperfect mimicry could arise due to the lack of improved benefits weighed against the costs of developing even more precise mimicry [22]. When models are relatively abundant, there are benefits for mimics to bear even a small resemblance to these models to avoid predation [23]. However, mimics are unlikely to improve their mimicry beyond the discriminability limits of predator vision due to costs to their fitness. Examples of such costs include reduced recognition by conspecifics (and hence lost mating opportunities) [24], reduced survivability in more heterogenous environments [18], and even less effective thermoregulation [25]. These trade-offs can be observed not only in lepidopteran species (such as *Papilio polytes* [26] and the Müllerian mimicry between viceroy and monarch butterflies [27]), but also in other insects such as hoverflies mimicking wasps [25], in ant-mimicking spiders [18], and may also play a role in mimicry between fish species [28]. Overall, the balance of costs and benefits suggests that mimics will evolve similar colour patterns to the models that protect them from predator attacks. However, the nature of this similarity, and specifically whether it relies more on similar colours or similar patterns, remains unclear. 

Here, we were interested in understanding the extent to which mimicry relies on similar patterns, rather than similar colours, in providing protection to prey. Similar colour patterns may arise convergently due to similar developmental mechanisms [29,30]. Such developmental biases, rather than natural selection imposed by predators, could explain some of the similarities observed between models and mimics, especially when species are closely related. Experiments involving pattern manipulations, however, can overcome these biases and test the limits of predator vision. Here, we use such manipulations to investigate how various pattern variants of a classic Batesian mimic affect its predation risk in the wild. 

Our study is focused on a well-known Batesian butterfly mimicry system [9,14] in which the unpalatable Common Rose (*Pachliopta aristolochiae*) is the aposematic model species with a “bright red body and red spots on the hindwings” [31] that advertises its toxicity. This toxicity is attributed to the presence of aristolochic acids and other alkaloids in their tissue [32,33] derived from the consumption of its host plant, the Dutchman’s Pipe (*Aristolochia tagala*), during its larval stages [33]. Its known mimic is the Common Mormon (*Papilio polytes*), which adopts a visually similar appearance without possessing the same toxicity. *Papilio polytes* exhibits female-limited sexual dimorphism, where only the *polytes* morph exhibits mimicry to *P. aristolochiae* [31]. This morph is an imperfect mimic of *P. aristolochiae*. It has a similar central white pattern and red dorsal marginal hindwing spots but does not have the same pink body colouration [31]. Despite some colour pattern differences, the model still gains strong protection against predators [14]. While many studies have investigated the evolutionary [34,35] and genetic bases [36,37] for this mimicry, the extent to which *P. polytes* can differ in its aposematic patterning from *P. aristolochiae* before losing its anti-predator function is not well understood. Focusing on this system, we investigated how patterns influence the efficacy of aposematic strategies and tried to identify some of the factors that allow for protection from predation despite imperfect mimicry.

**Figure 1:**
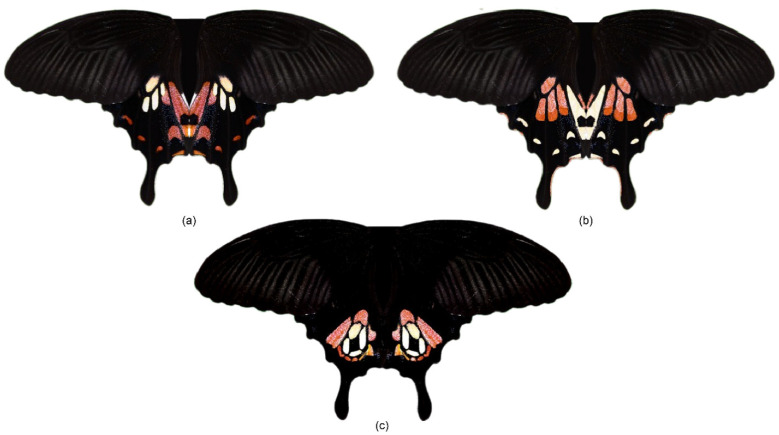
The three butterfly patterns deployed in this study. (**a**) *Papilio polytes* Wildtype pattern, (**b**) Flipped pattern, and (**c**) Eyespot pattern.

To address these aims, two modifications were made to the Wildtype (Wt) *P. polytes* pattern (Figure 1a). Firstly, a more minor modification was made by exchanging the positions of the red and white colours on the Wt hindwing whilst maintaining the positions of the patches themselves (Figure 1b). This was inspired by the imperfect, yet effective mimicry of the venomous Eastern Coral Snake (*Micrurus fulvius*) by the non-venomous Scarlet Kingsnake (*Lampropeltis elapsoides*) [13]. We hypothesised that this modification (hereinafter referred to as “Flipped”) would not significantly alter attack risk as compared to the Wt pattern, similar to the *M. fulvius*–*L. elapsoides* system. 

In the second—more major—modification (hereinafter referred to as the “Eyespot” modification), a large eyespot-like pattern was constructed by changing the original position of the pattern patches and rearranging them into an eyespot-like shape, with a black circle in the centre, encompassed by the white then the red regions (Figure 1c), whilst keeping the total area of red and white colours constant. Large eyespots, which can be quite conspicuous, are thought to deter predators through intimidation [38,39,40,41,42,43,44,45,46,47,48,49]. This intimidation hypothesis posits that large eyespots mimic vertebrate eyes that scare away potential predators [42,44,50,51]. We hypothesised that this major modification, which involves changing both the positions of the colours and the patches, would be a very imperfect Batesian mimic. However, two outcomes are possible. First, these models might have a significantly lower attack risk than the Wt models due to the potentially intimidating effect of the new eyespot-like pattern. Alternatively, this new Eyespot pattern might not be recognised as a mimic and hence experience higher predation relative to the Wt and Flipped patterns. 

## 2. Materials and Methods

In this study, Wt and modified (Flipped and Eyespot) paper butterfly models of the *P. polytes polytes* morph were created and deployed at three sites in Singapore as in previous studies [14,52,53]. The survival of the models was observed and plotted to measure their ability to deter predation attempts.

### 2.1. Preparation of Paper Butterfly Models

Three types of models were created: (1) unmodified Wt *polytes* morph, (2) Flipped type with a change in the position of colours BUT retaining the position of pattern patches, (3) Eyespot modification with a change in the position of colours AND position of pattern patches, where the pattern patches were arranged into an eyespot-like pattern (Figure 1). Firstly, a high-resolution image of *P. polytes* [54] was uploaded onto Adobe Photoshop 2023. The histogram function was used to quantify the number of pixels of specific colour regions on the hindwings in the image. In subsequent modifications, the number of pixels of each colour was kept constant. For the Flipped modification, the quick selection tool was used to select the white pattern patch on the left hindwing of the *polytes* morph (Figure 1a). Afterwards, using the clone stamp tool, the red colour was copied into the selected area. As there are more red than white pixels, to ensure that the total number of pixels per colour was kept the same as the original, the pattern patches that were originally white were slightly enlarged while retaining their original shape. The pattern patches that were originally red were then filled in with a white colour and the patch shrunk slightly while retaining its original shape. Afterwards, each colour was adjusted to resemble the reflectance spectra of the corresponding colours on real *P. polytes* wings (as described below). For the Eyespot modification, the quick selection tool was used to select each white or red pattern patch. They were then arranged into a single large eyespot in the middle of the hindwing. As much as possible, the original orientation of the patches was preserved. However, small rotational adjustments were made to ensure that the patches wrapped inwards towards each other. Based on observations of the Wt *polytes* morph, we decided to retain a black “iris” in the centre of the eyespot. As there were more red than white pixels, the white pixels were placed on the inside, while the red pixels were placed around them. 

**Figure 2:**
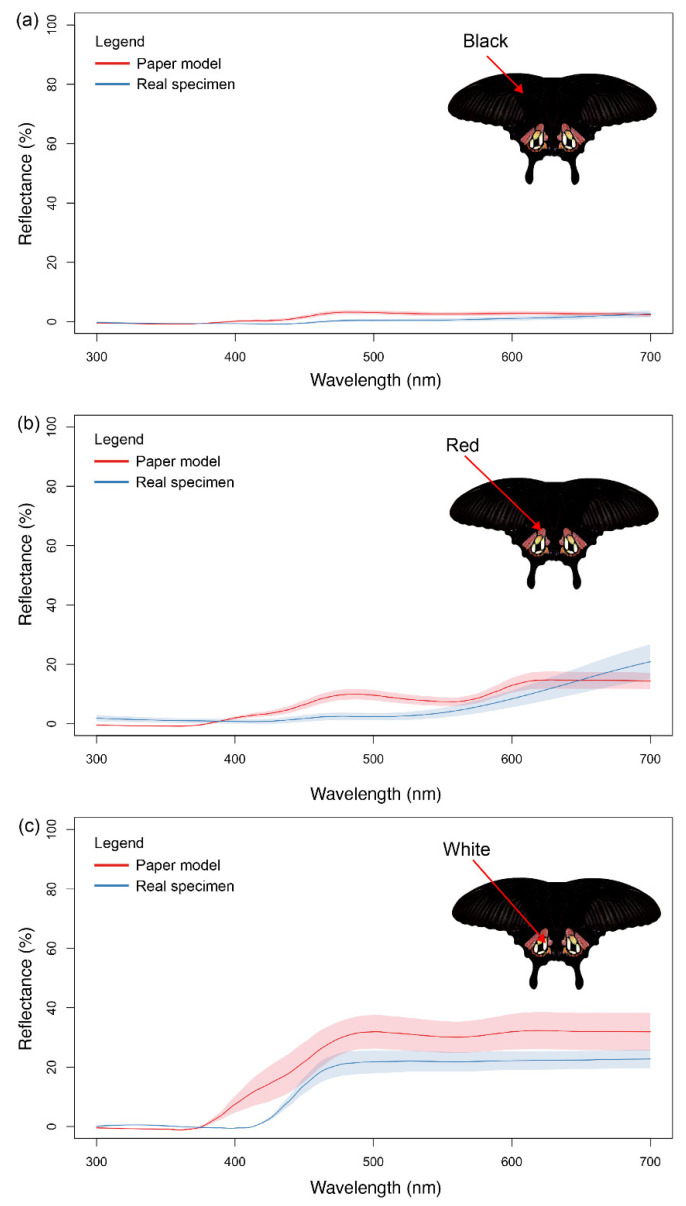
Plots of mean smoothed reflectance spectra of the colours on the dorsal side of real and artificial (paper) models of *P. polytes*. The Eyespot model is represented but similar reflectance spectra were obtained for the Flipped models (Supplementary Figures S1 and S2). The lines represent the mean, and the shaded areas represent the standard deviation of the spectral data (six measurements per colour were taken). (**a**) Black reflectance spectra, (**b**) red reflectance spectra, and (**c**) white reflectance spectra.

**Figure 3:**
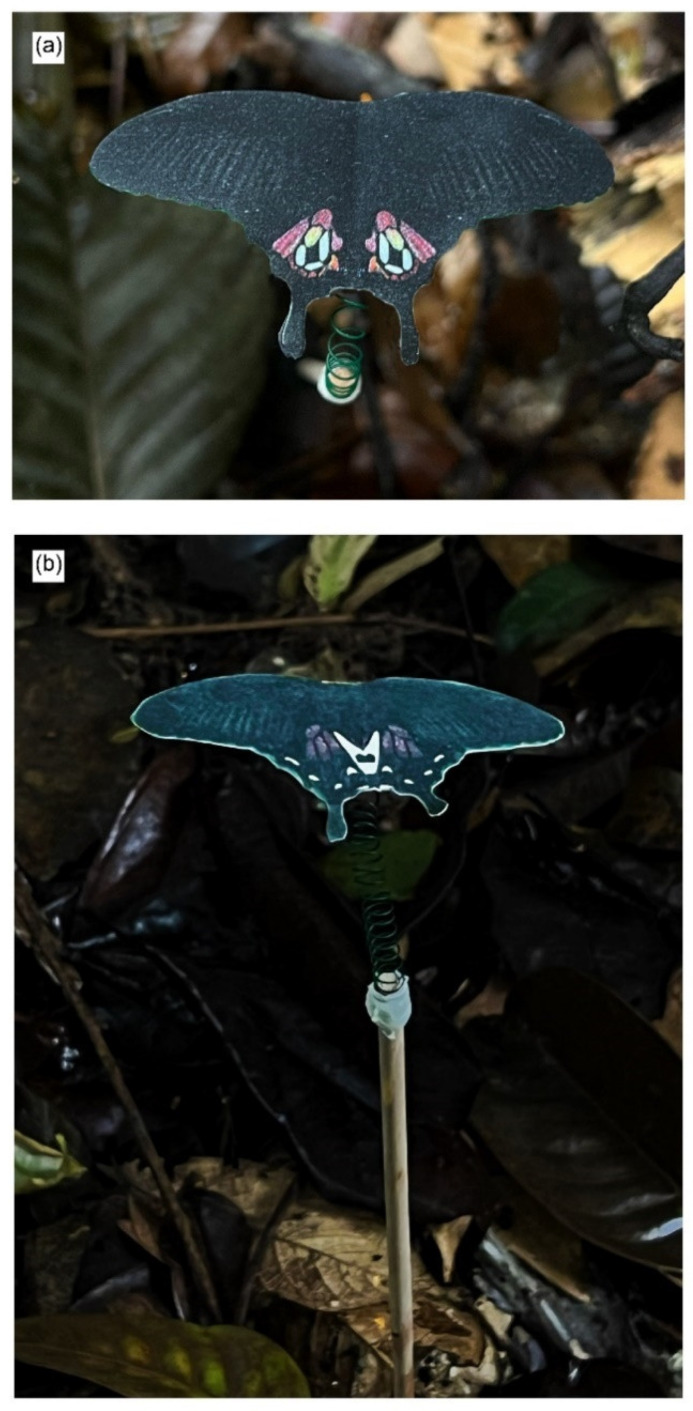
An example of our model setup. The butterfly paper models are suspended on a wire above a stick so that the paper models flit in the wind. (**a**) Top view. (**b**) Side view.

The model wings were printed on low-reflective Whatman qualitative filter paper (No. 1001-917) using a Canon ImageCLASS MF635Cx printer (Canon Singapore Pte. Ltd., Singapore) with CART045H toner in Black, Cyan, Magenta and Yellow. The printed model wings were cut out and affixed to pieces of black paper of 80gsm. The entire model (including the black backing) was sprayed with Krylon Preserve It! and Liquitex professional matte varnish water-resistant spray and allowed to dry. Afterwards, spectral measurements were obtained for the paper butterfly wings to ensure similarity to the real wings of *P. polytes* (Figure 2, Supplementary Figures S1 and S2). Wing specimens of *P. polytes* were obtained from a previous experiment [55]. An Ocean Optics USD2000 fibre optic spectrometer was used to take reflectance spectra measurements for the colours white, red, and black. The spectrometer was first calibrated using a white Ocean Optics WS-1 reflectance standard. Colours were adjusted as necessary, and the process was repeated, until the colours of the models resembled the colours in the live butterflies. The paper butterflies were attached to the free end of a wire, with the other end coiled around a wooden stick so that the paper models could flit in the wind. A live mealworm was attached to the central abdominal region on the ventral side of the patterns with liquid glue (UHU Multi-Purpose Adhesive) and clear tape (Scotch). A strip of Blu-Tack was then affixed over the mealworm so that any beak marks could be more easily visualised (Figure 3). A sponge soaked in insecticide was also affixed to the wooden stick about 15 cm above the ground to deter predation attempts by non-visual predators such as ants.

### 2.2. Deployment and Surveyance of Paper Models

Field experiments were carried out in Singapore in three different forested regions: MacRitchie Reservoir, Clementi Forest, and Kent Ridge Park. These locations were chosen because the host plants of *P. arisotolochiae* (i.e. *Aristolochia acuminata* and *Aristolochia ringens*) are found there and/or both our butterfly species of interest, *P. polytes* and *P. aristolochiae*, have been recorded there.

**Figure 4:**
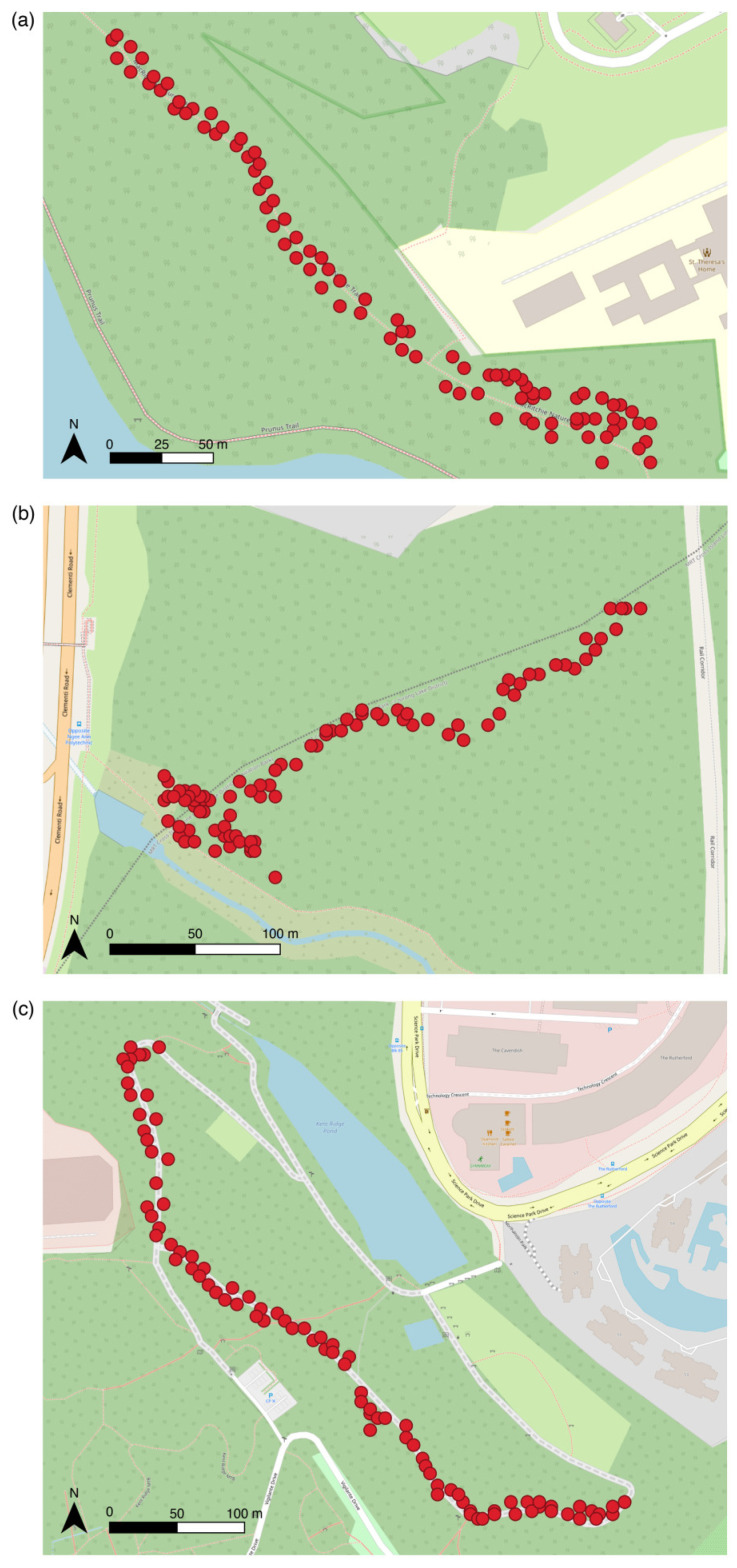
Individual points represent where each of the paper butterflies were deployed at each site in Singapore. (**a**) MacRitchie Reservoir Park (1.3488° N, 103.8224° E). (**b**) Clementi Forest (1.3294° N, 103.7802° E). (**c**) Kent Ridge Park (1.2939° N, 103.7913° E).

A total of 90 models were deployed per location (30 Wildtype, 30 Flipped, and 30 Eyespot). In total, 270 paper models were deployed across all three sites. The GPS coordinates and detailed locations of models at each site can be found in Figure 4. The *sample* function in R was used to randomly generate a sequence of deployment, and the 90 models were deployed according to the random sequence along the left and right sides of a 450 m transect line at each site. The paper models were placed 25–30 cm above the ground. There was a distance of at least five metres between each model.

**Figure 5:**
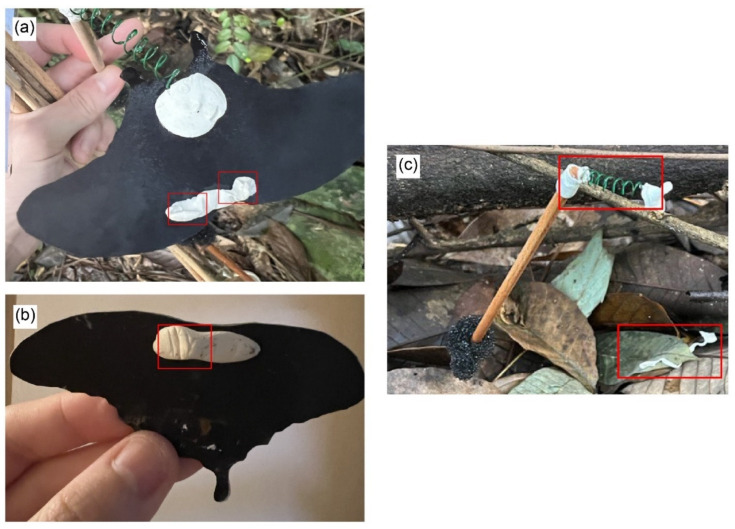
Bite marks or obvious tugging on the models (with or without mealworm present) were regarded as predation. (**a**,**b**) Red rectangles show distinct “V” shaped marks resembling those made by bird beaks. (**c**) The green wire has been pulled (top rectangle) and a long shred of Blu-Tack on the ground (bottom rectangle).

After the initial deployment, the models were checked every 24 h for signs of predation. Predation was defined as the absence of a mealworm, obvious beak markings (with or without worm present) or obvious tugging by predators (with or without worm present) (Figure 5). When the models were deemed to have been preyed upon, they were removed. Once 50% predation was obtained across a site, the experiment was stopped at that site. 

### 2.3. Statistical Analysis

All data analysis was carried out in R Studio v 4.1.3 [56]. A parametric survival analysis using the *survival* package [57,58] was conducted to test whether there was a significant difference in survival duration between the three types of models. Site was included as a random effect in our analysis. For models that were eaten by ants (i.e., ants were observed on the model or there was distinctive shredding of Blu-Tack into small pieces, in contrast to observations as detailed in Figure 5), the points were censored (i.e., coded as “0” in the dataset, whereas datapoints which were not censored were coded as “1”) before the analysis was run. 

## 3. Results

Of the 270 models deployed in total, 140 models were preyed upon. Of these 140, 38 were Wt models, 50 were Flipped models, and 52 were Eyespot models. Some models were attacked, and the mealworms plucked out, leaving behind visible triangular shaped beak imprints (Figure 5). For a few models, the paper models had been pulled, evidenced by the wire having been stretched out. In some models, Blu-Tack which was used to attach the mealworms was also torn into large shreds.

**Figure 6:**
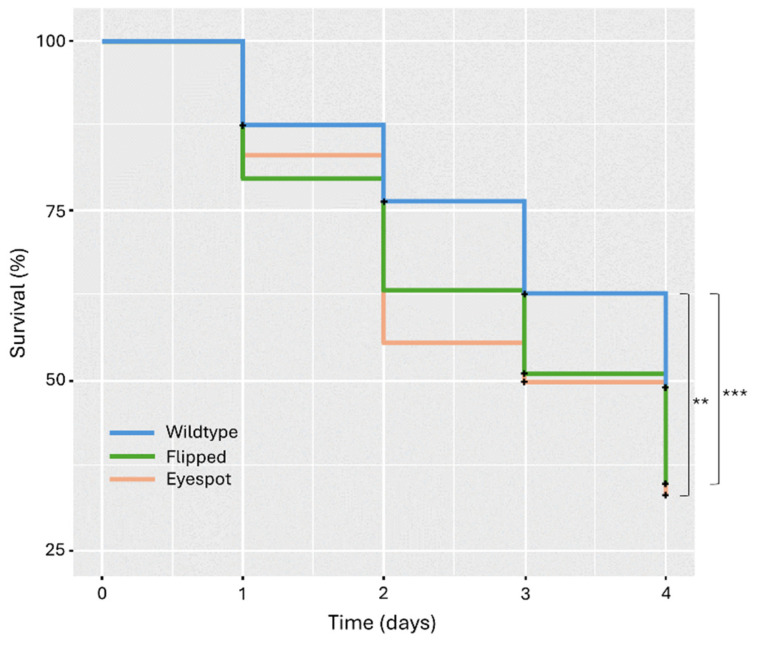
Survival curves showing the number of days that the models of each type survived in the field before being attacked. On average, the Eyespot types survived for the shortest number of days, followed by Flipped types, and finally, the Wildtype. Black crosses represent censored datapoints. **: *p*-value < 0.005, ***: *p*-value < 0.001.

Our results showed that the proportion of surviving models was highest for Wt (57%), followed by the Flipped type (44%) and finally the Eyespot type (42.2%). The Wt had a significantly higher survival than the Flipped type (*p*-value < 0.001) and the Eyespot type (*p*-value = 0.0024). There was no significant difference between the Flipped and Eyespot types (*p*-value = 0.69) (Figure 6). These results were consistent across all three sites (Supplementary Figure S3). 

## 4. Discussion

Batesian mimicry describes a situation where one, often palatable, species resembles another aposematic species in order to gain protection against predators. Whilst research has shown the importance of the mimic having similar colours as the model, less attention has been paid to whether it is important for the mimic to have a similar spatial pattern, i.e., the location of the colours on the body. Using paper models of *Papilio polytes* butterflies (which are palatable mimics of an aposematic species, *Pachliopta aristolochiae*) deployed in the field, we tested how two modifications of the pattern—one minor and one relatively major—affected predation rates. The results show that pattern matters and predators are able to distinguish between the Wt and the two pattern modifications. 

Both the Flipped and the Eyespot models were attacked more frequently than Wt models. This is contradictory to one of our initial hypotheses. We had hypothesised that predators would not be able to distinguish the Flipped pattern from the Wt pattern, but this minor modification was distinguishable by predators, and these Flipped models were attacked more than Wt models. The higher predation on both types of models, relative to Wt, is likely because they were poorer mimics of the distasteful model, *P. aristolochiae*, suggesting that predators are able to distinguish between animals based on where colours are located on their bodies. The increased survival of the Wt models is likely due to some natural predators having prior exposure to the toxic *P. aristolochiae* (the aposematic model species), resulting in avoidance of the *P. polytes* Wt models, a well-established successful mimic [9,14,31]. 

The decrease in attacks on Flipped models as compared to the Wt models illustrates that the preservation of the overall position of pattern patches is not sufficient to fool predators. This result suggests that predators associate specific bright colours to specific positions during the “learning” process of avoiding unpalatable or toxic species. Swapping the red and white colours appears to have resulted in less effective mimicry of *P. aristolochiae* by the Flipped-type paper models. Similarly, in a previous study by De Bona et al. [50], a swapping of colours was performed within the eyespots of another butterfly species, *Caligo martia*. Their results showed that the flipped colouration resulted in a significantly lower aversion by predators as opposed to the original eyespot. Our results provide further support for the idea that predators are deterred by the association of certain colours to specific positions in aposematic strategies. 

This finding contrasts with the *M. fulvius–L. elapsoides* (snake) system, where the pattern of the *L. elapsoides* mimic, despite having a flipped order in its colour stripes relative to the venomous *M. fulvius* model, was still protective. This suggests that the degree to which patterns can deviate from a model pattern varies across systems. Various factors affect the response of predators to prey, such as distance at which they attack [59], the different visual acuity of different predators [60,61], the type of visual pigments in their eyes [62], and whether the prey displays movement (e.g., Prudic et al. [63] describe this for praying mantids; they only attack after a butterfly makes a movement). Snakes and butterflies are likely to be preyed upon by different species, and one or more of these factors may explain the differences between the two systems.

It was interesting to see that the Eyespot models also had a decreased survival as compared to Wt models. This suggests that the eyespot-like pattern created did not intimidate the predators in our study. A possible explanation for this is the small size of the eyespot-like pattern relative to overall wing size in our paper models. Previous experiments showed that a single large eyespot on the wings of a non-toxic species (*Mycalesis perseus*) was more intimidating to predators relative to either models without eyespots or models with smaller eyespots [47]. The intimidating eyespots in Ho et al. [47] were only 6 mm in diameter, whereas the eyespots in our study averaged 7.5–11 mm in diameter (Supplementary Table S1). If absolute eyespot size is the only factor that determines whether an eyespot is intimidating [40,50], the current eyespots should exert a strong intimidation effect. However, the ratio of eyespot size to wing size is lower in the current study (0.0290–0.0797) relative to that in Ho et al. [47] (0.156–0.264; Supplementary Table S1), and in the Ho et al. study, when eyespot size relative to wing size decreased (via an increase in wing size, keeping eyespot size constant), there was a slight increase in predation. This suggests that if predators evaluate relative instead of absolute eyespot sizes, then the Eyespot models could function instead to attract predators [47]. The exact reason of why the eyespot models did not perform as well as (or better than) the Wt models needs additional study, where different factors are isolated in turn, although it should be considered that some of the factors in the patterns may not be important for mimicry in the eyes of predators [64]. Overall, our results suggest that large eyespots are less intimidating to predators than aposematic patterns that are better mimics of a toxic species. 

The predation rates we measured for the Eyespot and Flipped patterns may have been underestimated relative to the Wt patterns due to neophobia or dietary conservatism [65,66,67,68,69,70,71]. While naïve predators, i.e., those which have never encountered *P. aristolochiae* or *P. polytes*, would likely have exhibited neophobia or dietary conservatism towards all three types of models equally, experienced predators might have avoided the Flipped- and Eyespot-type models only, as these represented wing patterns they encountered for the first time. These two model types also do not resemble any species, palatable or otherwise, found in Singapore. Hence, the higher predation rates we observed on these two types of models may, thus, be an underestimation of their ineffective mimicry.

Finally, whilst we could not ascertain the species of avian predators attacking the different patterns in this study, avian predators are known to cross into different areas to forage [72], and it is likely that, at the distances involved, the three deployed patterns were encountered by the same few species of birds such as the Javan myna *Acridotheres javanicus*, Asian koel *Eudynamys scolopaceus,* and the black-naped oriole *Oriolus chinensis*. Nevertheless, it would be interesting for a future experiment to try to identify the predators attacking the models via camera traps. Overall, this study contributes to our understanding of aposematic colouration by showing that pattern is important and that relatively minor modifications to the patterns of *P. polytes* make it an imperfect mimic of *P. aristolochiae* in Singapore forests. 

## Data Availability

The original contributions presented in the study are included in the Supplementary Materials.

## Supplementary Materials

The following supporting information can be downloaded at: https://www.mdpi.com/article/10.3390/insects15070465/s1, Supplementary Figure S1: Plots of mean smoothed reflectance spectra of the colours on the dorsal side of real and artificial (paper) Flipped *P. polytes*; Supplementary Figure S2: Plots of mean smoothed reflectance spectra of the colours on the dorsal side of real and artificial (paper) Wildtype *P. polytes* dorsal side; Supplementary Table S1: Proportion of eyespot area relative to wing area from Ho et al. [47] and this study was determined using an online software program (SketchandCalc); Supplementary Figure S3: Survival curves showing the number of days that the models of each type survived in the field before being attacked per site. Survival analysis dataset: Lim et al – Data.xlsx; and Survival analysis code and metadata: Lim et al – Rcode and Metadata.R.
