# Supplementary material for: Pattern Matters in the Aposematic Colouration of Papilio polytes Butterflies
Source: Insects. 2024 Jun 22;15(7):465. doi: 10.3390/insects15070465 (PMC11277510; doi:10.3390/insects15070465)
Supplement: Supplementary file 1 [file insects-15-00465-s001.zip › Lim et al - Supplementary Figures and Tables.pdf]

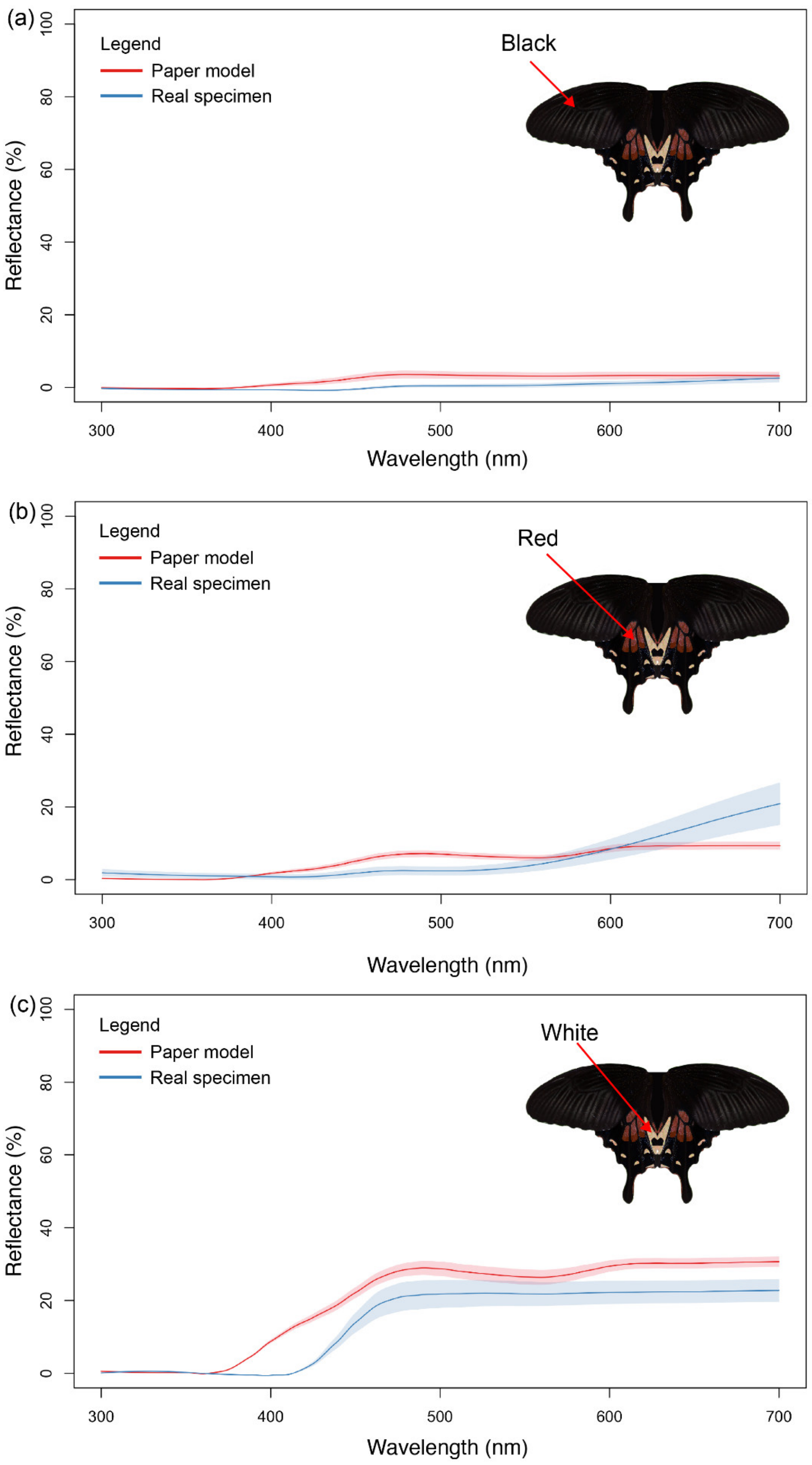

Supplementary Figure S1. Plots of mean smoothed reflectance spectra of the colours on the dorsal side of real and artificial (paper) Flipped *P. polytes*. The lines represent the mean, and the shaded areas represent the standard deviation of the spectral data (n=6 for each type). (a) Black reflectance spectra, (b) Red reflectance spectra and (c) White reflectance spectra.

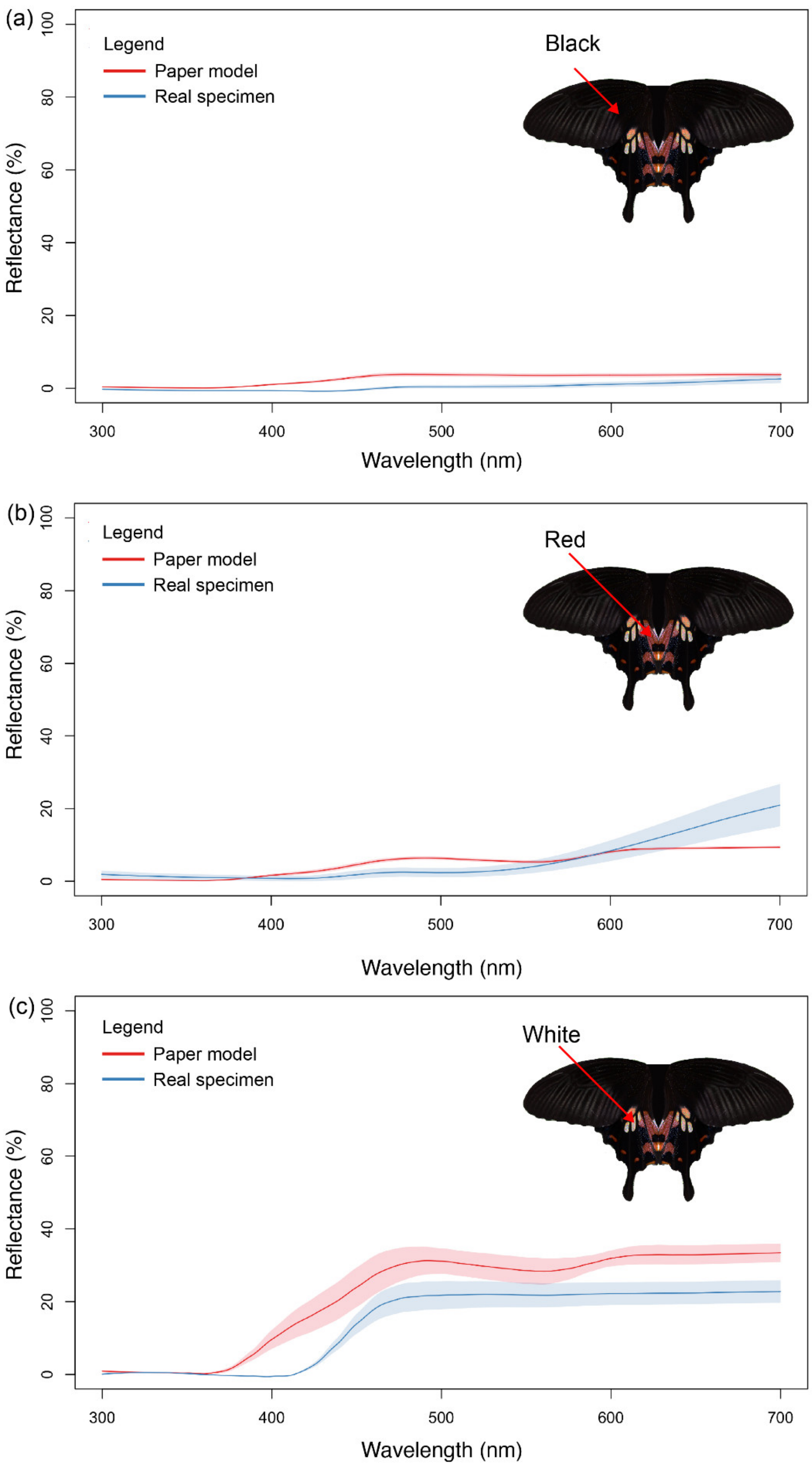

Supplementary Figure S2. Plots of mean smoothed reflectance spectra of the colours on the dorsal side of real and artificial (paper) Wildtype *P. polytes* dorsal side. The lines represent the mean, and the shaded areas represent the standard deviation of the spectral data (n=6 for each type). (a) Black reflectance spectra, (b) Red reflectance spectra and (c) White reflectance spectra.

Supplementary Table S1. Proportion of eyespot area relative to wing area from Ho et al. [47] and this study was determined using an online software program (SketchandCalc). The proportion of eyespot area in this study falls below the proportion of eyespot area corresponding to an eyespot size of 6 mm from Ho et al. [47].

Study	Ho et al. [47]				This study	
Reference model	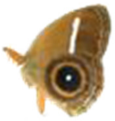 Experiment 3	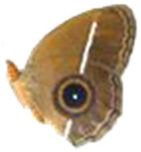 Experiment 5	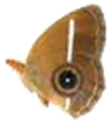 Experiment 4	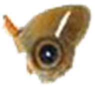 Experiment 6	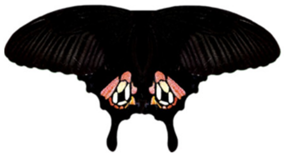	
Eyespot diameter (mm)	8	8	6	6	10-12	7-8
Description	Model with single large eyespot of 8mm	Model with eyespot of 8mm with enlarged wings	Model with single eyespot of 6mm	Model with eyespot of 6mm and reduced wings	Eyespot up to red margins	Eyespot up to white margins
Area of eyespot (mm^2)	50.27	50.27	28.27	28.27	83.96	30.6
Area of right side of hind- and forewing	190.36	322.95	208.9	127.64	1053.4	1053.4
Proportion	0.264	0.156	0.135	0.221	0.0797	0.0290

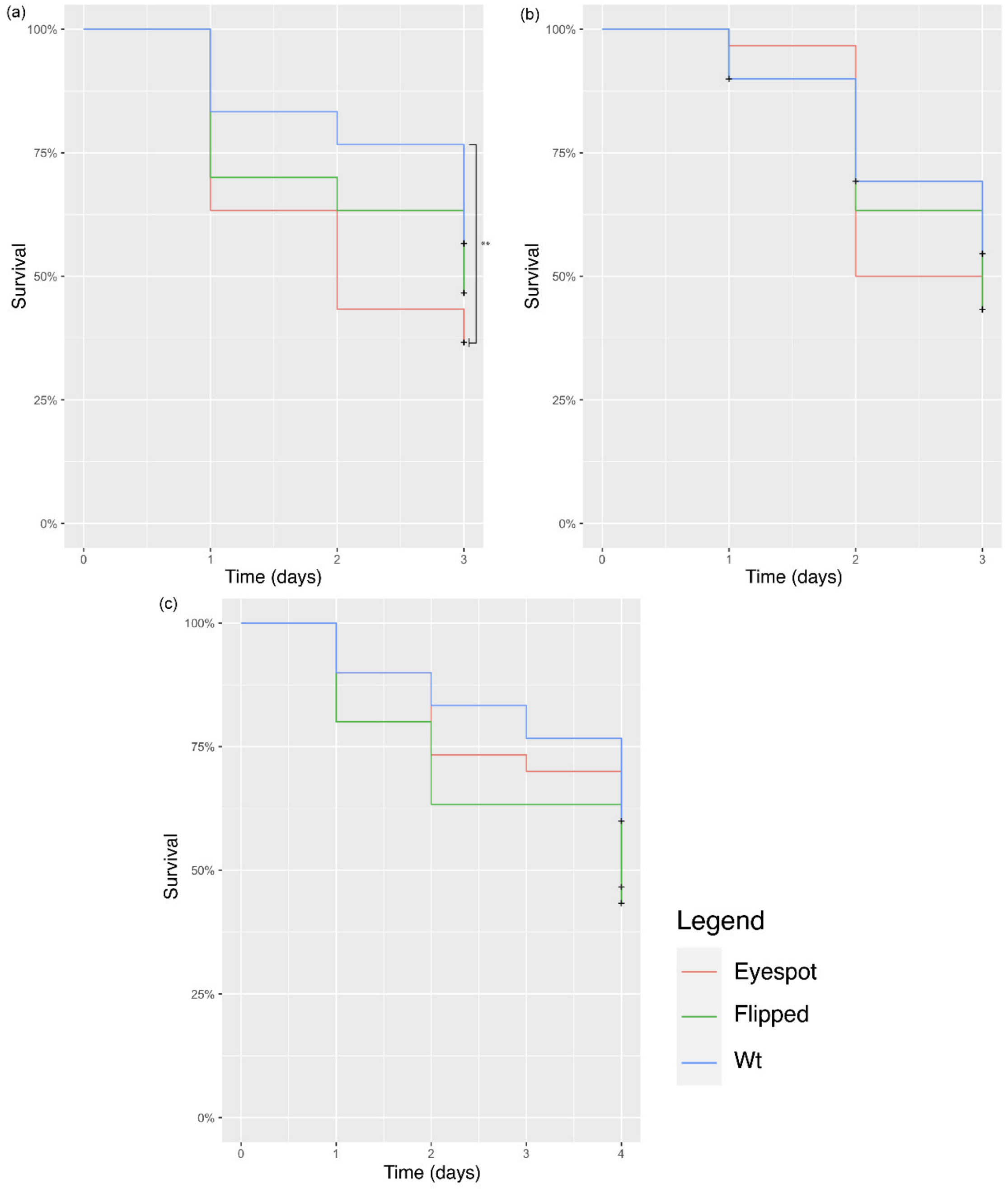

Supplementary Figure S3. Survival curves showing the number of days that the models of each type survived in the field before being attacked per site. (a) MacRitchie Reservoir Park. (b) Clementi Forest. (c) Kent Ridge Park. Across all three sites, Wt models consistently had the highest survival rates, and the survival rates of Eyespot and Flipped models were not significantly different from each other. The difference between Wt vs. Flipped and Wt vs. Eyespot models is significant when all sites are analysed together (whilst accounting for amongst-site differences in the survival analysis). **:p-value = 0.02.
